# Colitis Induces Sex-Specific Intestinal Transcriptomic Responses in Mice

**DOI:** 10.3390/ijms231810408

**Published:** 2022-09-08

**Authors:** Linnea Hases, Madeleine Birgersson, Rajitha Indukuri, Amena Archer, Cecilia Williams

**Affiliations:** 1 Science for Life Laboratory, Department of Protein Science, KTH Royal Institute of Technology, 171 21 Solna, Sweden; 2 Department of Biosciences and Nutrition, Karolinska Institutet, 141 83 Huddinge, Sweden

**Keywords:** colitis, CAC, mouse model, AOM/DSS, sex differences, transcriptome

## Abstract

There are significant sex differences in colorectal cancer (CRC), including in incidence, onset, and molecular characteristics. Further, while inflammatory bowel disease (IBD) is a risk factor for CRC in both sexes, men with IBD have a 60% higher risk of developing CRC compared to women. In this study, we investigated sex differences during colitis-associated CRC (CAC) using a chemically induced CAC mouse model. The mice were treated with azoxymethane (AOM) and dextran sodium sulfate (DSS) and followed for 9 and 15 weeks. We performed RNA-sequencing of colon samples from males (*n* = 15) and females (*n* = 15) to study different stages of inflammation and identify corresponding transcriptomic sex differences in non-tumor colon tissue. We found a significant transcriptome response to AOM/DSS treatment in both sexes, including in pathways related to inflammation and cell proliferation. Notably, we found a stronger response in males and that male-specific differentially expressed genes were involved in NFκB signaling and circadian rhythm. Further, an overrepresented proportion of male-specific gene regulations were predicted to be targets of Stat3, whereas for females, targets of the glucocorticoid receptor (Gr/Nr3c1) were overrepresented. At 15 weeks, the most apparent sex difference involved genes with functions in T cell proliferation, followed by the regulation of demethylases. The majority of sex differences were thus related to inflammation and the immune system. Our novel data, profiling the transcriptomic response to chemically induced colitis and CAC, indicate clear sex differences in CRC initiation and progression.

## 1. Introduction

Tumor development is pronounced in patients with inflammatory bowel disease (IBD) and chronic inflammation is one of the general hallmarks of colorectal cancer (CRC) [1,2,3]. IBD-CRC follows the inflammation-dysplasia-carcinoma sequence [4], and although IBD is a risk factor for CRC in both sexes, the risk of developing CRC is 60% higher for male IBD patients than for female [5]. Worldwide, CRC is the third most common cancer in both sexes combined [6], and better preventative and treatment strategies are needed to reduce the mortality rates. Interestingly, significant sex differences are present in CRC [7,8]. Men have a higher incidence and earlier onset of CRC compared to women [7,9], and a lower overall survival compared to age-matched premenopausal women. Men are more prone to left-sided CRC, whereas women are more prone to right-sided CRC [7]. Male tumors more often have *NRAS* mutations, whereas tumors from women more often exhibit *BRAF* mutations and microsatellite instability (MSI) [10]. Recently, we also found that several prognostic biomarker candidates in CRC are sex-dependent [11].

A diet rich in fat is known to induce a low-grade inflammatory state of the colon and is considered a risk factor for CRC development. We have previously demonstrated sex differences in the colon and its transcriptome in mice fed a high-fat diet (HFD) and observed differences in crypt proliferation and immune cell infiltration, along with differences in the expression of circadian clock genes [12]. Colitis increases the risk of CRC, and the importance of histologic inflammation over time has been highlighted as an important risk factor for colitis-associated cancer (CAC) [13]. A well-established mouse model to study CAC is the azoxymethane (AOM)/dextran sulfate sodium (DSS) model, which closely resembles human CAC. Multiple studies have investigated the transcriptomic changes during AOM/DSS-induced colitis and CAC to identify novel clinical candidates in the pathogenesis of human CAC. However, few studies have focused on identifying sex-specific gene targets, despite the overwhelming sex disparities seen in CRC. To our knowledge, no studies have previously investigated sex differences in the progression of CAC.

Here, we aimed to explore the molecular underpinnings of the phenotypic sex differences previously noted, including the higher propensity of males for colitis and subsequent tumor development. We hypothesized that female and male mice respond differently to inflammation at the transcriptional level and that this would include specific signaling pathways. To characterize these differences, and the key regulatory factors, we investigated the transcriptomes of the colonic epithelial layer of mice of both sexes at two time points (9 and 15 weeks) following initiation of AOM/DSS treatment. The overarching goal of this study was to improve the understanding of sex differences in disease initiation and progression.

## 2. Results

**Figure 1:**
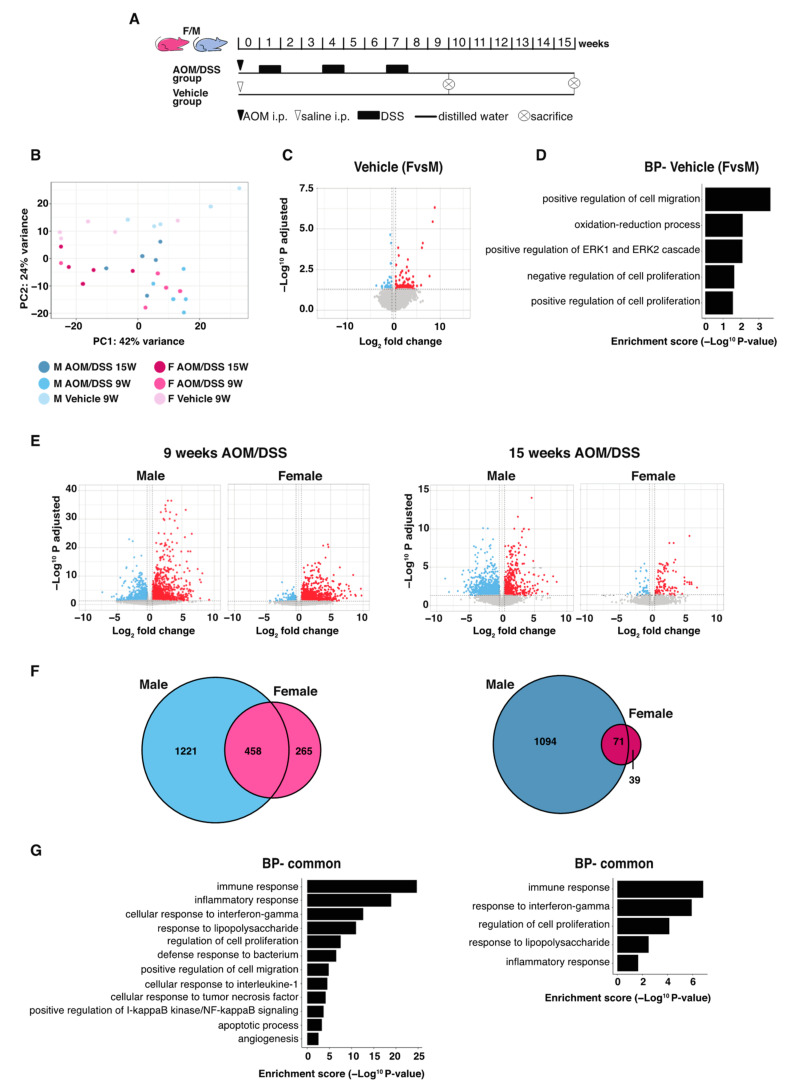
Sex differences in the colon transcriptome. (**A**) Experimental setup: male and female mice underwent treatment with vehicle or AOM/DSS for 9 or 15 weeks. (**B**) PCA plot of vehicle- and AOM/DSS-treated (9 and 15 weeks) male (blue) and female (pink) mice. (**C**) Volcano plot of differentially expressed genes (DEGs) between female and male mice during vehicle treatment. (**D**) BP enrichment analysis of the DEGs between females and males. (**E**) Volcano plots of DEGs between vehicle- and AOM/DSS-treated (9 and 15 weeks) for male (**left**) and female (**right**) mice. (**F**) Venn diagram showing that males responded more strongly to 9 and 15 weeks AOM/DSS treatment, with more DEGs identified, and that both common and sex-specific genes were identified. (**G**) BP enrichment analysis of the common DEGs during 9 (top) and 15 (bottom) weeks of AOM/DSS treatment.

In our previous animal study, we had noted sex differences following AOM/DSS-induced tumor formation, including larger tumors and higher expression of specific cytokines in males compared to females [14]. To identify the complete transcriptomic response to AOM/DSS and related sex differences in the colon, we sequenced RNA from the epithelial layer (scrape) from mice of both sexes treated with vehicle or AOM/DSS for 9 and 15 weeks, respectively (Figure 1A). The 9-week time point is soon after the third DSS (intestinal irritant) cycle, where mice exhibit colitis in several areas of their intestines [14], and we here denote this state as a more acute phase of colitis. At 15 weeks, nearly two months after the last DSS cycle, the inflammation is less pronounced (as previously reported [14]), and we here denote this stage as a more chronic state of inflammation. At this stage, all mice had developed tumors, and male mice developed larger tumors than females [14].

### 2.1. Sex Differences Are Apparent in the Colon Transcriptome

When comparing normal (intraperitoneal injection, i.p., with saline vehicle) male and female colon epithelial transcriptomes in a principal component analysis (PCA), we found that the sexes clustered separately (Figure 1B, light pink versus light blue). The transcriptomic signal-to-noise ratio was above one (tSNR > 1), which supported a high overall divergence of transcriptomes between males and females (Figure S1D). The majority of the DEGs between the sexes in the colon were upregulated in males (Figure 1C and Figure S1C, Table S1). Overall, the DEGs that differed between the sexes were enriched for functions and pathways related to cell proliferation, cell migration, and oxidation-reduction process (Figure 1D).

### 2.2. AOM/DSS Treatment Induces an Immune and Inflammatory Response in Both Sexes

To investigate how AOM/DSS-induced colitis affects the mouse colon epithelial transcriptome after 9 and 15 weeks of treatment, we compared the colon epithelial gene expression of vehicle-treated mice to AOM/DSS-treated mice. We found a clear separation between vehicle- and AOM/DSS-treated mice by PCA (both sexes) and identified a high transcriptomic divergence (tSNR > 1) (Figure 1B and Figure S1D). The volcano plots illustrate the DEGs per sex and time point, and the Venn diagrams show the overlap between sexes (Figure 1E,F and Figure S1C, and Tables S2 and S3). We note that a majority of the genes that responded to treatment in females also did so in males (458 of 728 or 63% at 9 weeks; 79 out of 110 or 72% at 15 weeks). Biological process (BP) enrichment analysis revealed that, at both time points, the genes responding to treatment in both sexes were involved in immune and inflammatory responses, regulation of cell proliferation, and response to lipopolysaccharide and interferon gamma (Figure 1G). In addition, at 9 weeks, apoptosis, response to bacterium, cellular response to TNF and interleukin-1, and positive regulation of NFκB signaling were enriched in both sexes (Figure 1G). This demonstrates expected changes and validates the methodological setup.

### 2.3. The Transcriptomic Response to Colitis Differs between Males and Females

**Figure 2:**
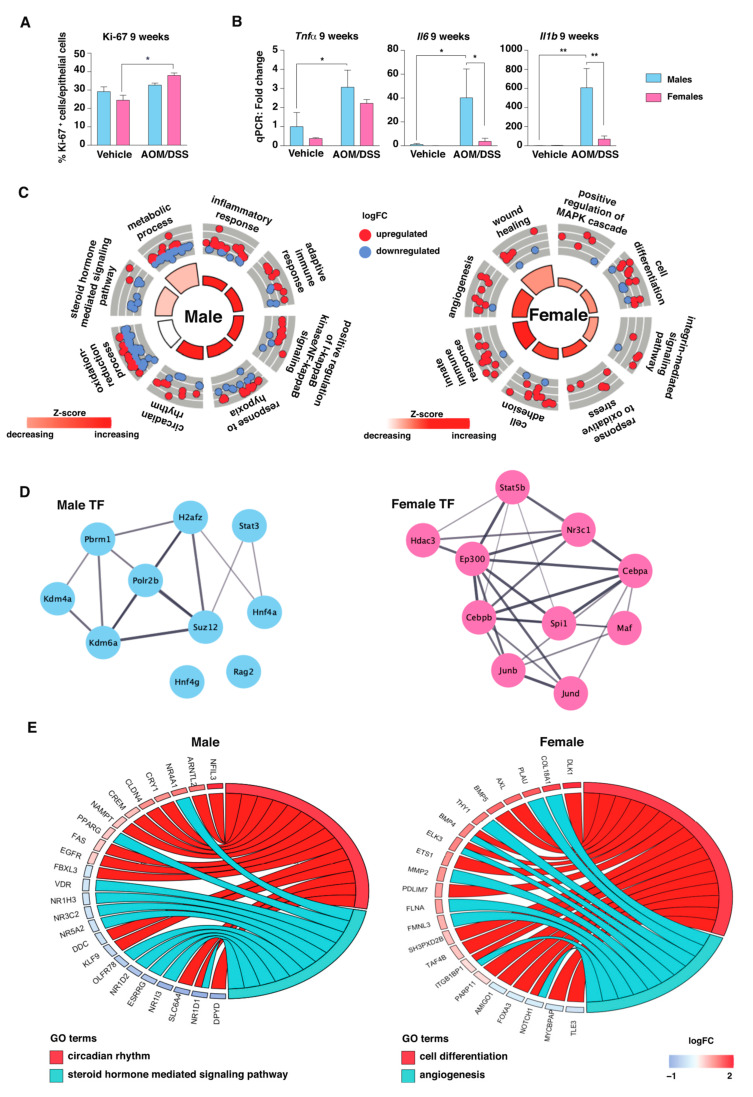
The sex-dependent response to 9 weeks AOM/DSS treatment. Comparison of (**A**) Ki-67 protein levels (immunohistochemical analysis) and (**B**) expression of the pro-inflammatory genes *Tnfα, Il6*, and *Il1b* (qPCR analysis), indicate sex differences in cell proliferation and inflammation between males and females, both based on data from [14]. (**C**) BP enrichment analysis of the sex-specific genes (RNA-seq), and (**D**) prediction of related transcription factor activity for male- and female-specific DEGs using BART. (**E**) Circular plots displaying the relationship between the genes that were differentially expressed between vehicle and 9 weeks of AOM/DSS treatment and the GO terms circadian rhythm and steroid hormone signaling in males (**left**) and cell differentiation and apoptosis in females (**right**), including fold changes (logFC) of corresponding up- and downregulated genes. Statistical analysis was performed using two-way ANOVA tests with Fisher’s LSD tests (* <0.05, ** <0.01).

We have previously identified that females presented a significant increase in colonic crypt cell proliferation after 9 weeks of AOM/DSS treatment, which was not seen in males (Figure 2A and [14]). Males, on the other hand, presented a significantly stronger increase of the pro-inflammatory cytokines *Tnfα*, *Il6*, and *Il1b* (Figure 2B). In order to comprehensively identify the general pattern of sex differences during this more acute phase of colitis (9 weeks after initiation of DSS treatment, and shortly after the final DSS cycle), we focused on the corresponding transcriptomes at this time point. The PCA plot shows that the separation between the sexes had decreased compared to their non-inflamed baseline level (vehicle-treated, Figure 1A), plausibly because of the common response to colitis in both sexes (Figure 1F, top). Still, when compared to respective vehicle-treatment within the sexes, male AOM/DSS-treated colon displayed a stronger response to the induction of colitis (1679 DEGs) compared to female (723 DEGs) (Figure 1E,F top, Figure S1C, Table S2) and more genes (1221) were uniquely regulated in males, compared to in females (225 genes, Figure 1E, top panel). The male-specific gene regulations were primarily involved in metabolic processes, hormone signaling (mostly downregulated), response to hypoxia, positive regulation of NFκB (mostly upregulated), and circadian rhythm (Figure 2C,E, left panels). Fourteen male-specific DEGs were involved in the circadian rhythm, including a downregulation of the nuclear receptor *Rev-erbα* (*Nr1d1*) and upregulation of the core clock genes *Cry1* and *Arntl2* (Figure 2E). Interestingly, 11 male-specific DEGs were involved in hormone signaling, and most were downregulated (Figure 2E, left panel). These included several nuclear receptors in addition to *Rev-erbα*, namely *Rev-erbβ* (*Nr1d2)*, the mineralocorticoid receptor (*Nr3c2*), liver receptor homolog-1 (*Lrh1/Nr5a2*), the oxysterol receptor Lxrα (*Nr1h3*), constitutive and rostane receptor (*Car/Nr1i3*), estrogen receptor-related receptor gamma (*Esrrg*), and vitamin D receptor (*Vdr*). Only two nuclear receptors were upregulated, namely peroxisome proliferator-activated receptor gamma (*Pparg*) and nerve growth factor IB (*Nur77/Nr4a1*). Nur77 is known to be expressed in macrophages and to play a role in inflammation. The female-specific pathways, on the other hand, were enriched for functions in angiogenesis, cell adhesion, cell differentiation, and response to oxidative stress (Figure 2C,E, right panels).

To predict which transcription factors were responsible for the noted sex-dependent gene regulation, we performed Binding Analysis for Regulation of Transcription (BART) analysis on the sex-specific DEGs. Interestingly, the top-10 transcription factors for male-specific DEGs included Stat3, a protein that is activated in response to various cytokines (including Il6), and two lysine demethylases (Kdm4a and Kdm6a) (Figure 2D). The predicted female-specific transcription factors included glucocorticoid receptor (Gr, Nr3c1), a key regulator of inflammatory responses, and two AP-1 family members, namely Jund and Junb (Figure 2D).

We have previously described sex differences in the colon during HFD [12], and that data set was simultaneously analyzed using BART here. Similarly, the HFD male-specific genes were also predicted to be regulated by Stat3 and the female-specific DEGs were predicted to be regulated by Gr (Nr3c1) (Figure S2A). Also, Cebpa and Cebpb, with important functions in regulation of Gr activity, were indicated in females. In conclusion, we found a sex-specific response to 9 weeks of AOM/DSS treatment, with males displaying a stronger transcriptomic response to the induction of colitis. Stat3 was indicated as a strong transcriptional activator in males, while Gr transcriptional targets were prominent among female-regulated genes.

### 2.4. Males Exhibit Stronger Long-Term Inflammatory Response during Tumor Development

**Figure 3:**
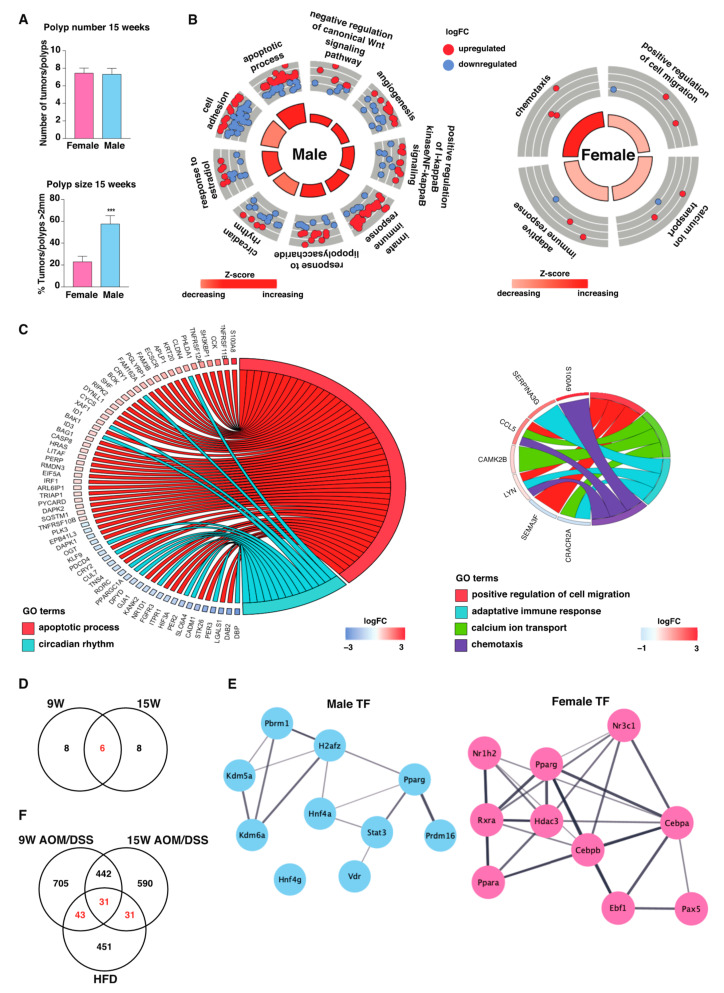
Males display a stronger response to 15 weeks of AOM/DSS treatment. (**A**) Comparison of number and size of polyps between males and females after 15 weeks of AOM/DSS treatment (data from [14]). (**B**) The BP enrichment analysis of the sex-specific DEGs. (**C**) Circular plot displaying the relationship between DEGs (vehicle compared to 15-week AOM/DSS treatment) and their functions (GO terms), apoptotic process and circadian rhythm in males (**left**) and calcium ion transport, cell migration, adaptive immune response, and chemotaxis in females (**right**), including enrichment (z score) and direction of corresponding regulated genes(+/−logFC). (**D**) Venn diagram showing the overlap between the male-specific DEGs involved in circadian rhythm during 9 and 15 weeks of treatment. (**E**) Transcription factors predicted (with BART) to regulate the male- and female-specific DEGs. (**F**) Venn diagram comparing the male-specific response after 9 and 15 weeks of treatment with HFD feeding (for 13 weeks, transcriptome from [12]). Statistical analysis was performed using the student’s *t*-test (*** <0.001).

After 15 weeks of AOM/DSS treatment, males had developed significantly larger tumors compared to females (Figure 3A, based on previously reported data [14]). In the colon epithelial transcriptome (not tumor), the separation between sexes was evident at this time point. The difference between vehicle-treated and AOM/DSS-treated mice was clearer and larger at the 15-week time point compared to the 9-week time point (Figure 2B). In males, up- and downregulated genes were equally distributed, while in females, most genes were upregulated (Figure 1D and Figure S1C). Again, males displayed a stronger transcriptomic response to treatment, with 1165 DEGs identified compared to 110 in females (Figure 1E and Figure S1C, Table S3), with few gene regulations being unique to females (39 genes, Figure 1E). Similar to the more acute state of colitis (9 weeks), the male-specific pathways during the more chronic state of colitis (15 weeks) were involved in circadian rhythm and positive regulation of NFκB, but also in the apoptotic process and the innate immune response (Figure 3B,C). Fourteen male-specific DEGs were involved in circadian rhythm, with six of these genes also found at 9 weeks (including downregulation of *Rev-erbα* and upregulation of *Cry1*, Figure 3C,D). Specific for the 15-week time point were downregulations of the core clock genes *Cry2*, *Per2*, and *Per3*, as well as the circadian-related gene RAR-related orphan receptor C (*Rorc*) (Figure 3C). The BART analysis again predicted Stat3 and two lysine demethylases (Kdm5a and Kdm6a) to be partially responsible for the male-specific DEGs, but also the nuclear receptors Pparg and Vdr (Figure 3E). Interestingly, when we compared the male-specific response to AOM/DSS treatment (9 and 15 weeks) with the previous analysis of male-specific response to HFD (from [12]), we found 105 genes in common (Figure 3F). Among the 31 common genes after 15 weeks of treatment, we identified the core clock gene *Per2* and the circadian-related gene *Rorc*. We have previously described sex differences in the human colon and CRC [11], and we now performed BART analysis on this data set, to see which transcription factors are predicted to be involved in human CRC tumorigenesis. Interestingly, we found that STAT3 was predicted to also regulate the male-specific transcriptome response (DEG between normal human colon and CRC, Figure S2B).

The fewer female-specific DEGs at 15 weeks (31 genes) were enriched for functions related to calcium ion transport, cell migration, adaptive immune response, and chemotaxis (Figure 3B,C, right panels). These were again predicted to be regulated by Gr, but also by other nuclear receptors including liver X receptor beta (Lxrβ/Nr1h2), Ppara, Pparg, and retinoid X receptor alpha (Rxra, Figure 3E).

Our data thus convincingly demonstrates that there is a sex-specific response to AOM/DSS. Males presented a more pronounced transcriptomic response also at 15 weeks, again with a sex-specific alteration of the circadian rhythm and a predicted sex-specific activation by Stat3, a transcription factor we here also predict as regulator for the male-specific response to HFD and male-specific gene expression in human colon.

### 2.5. Consistent Sex Differences Were Most Apparent at Chronic State of Inflammation

**Figure 4:**
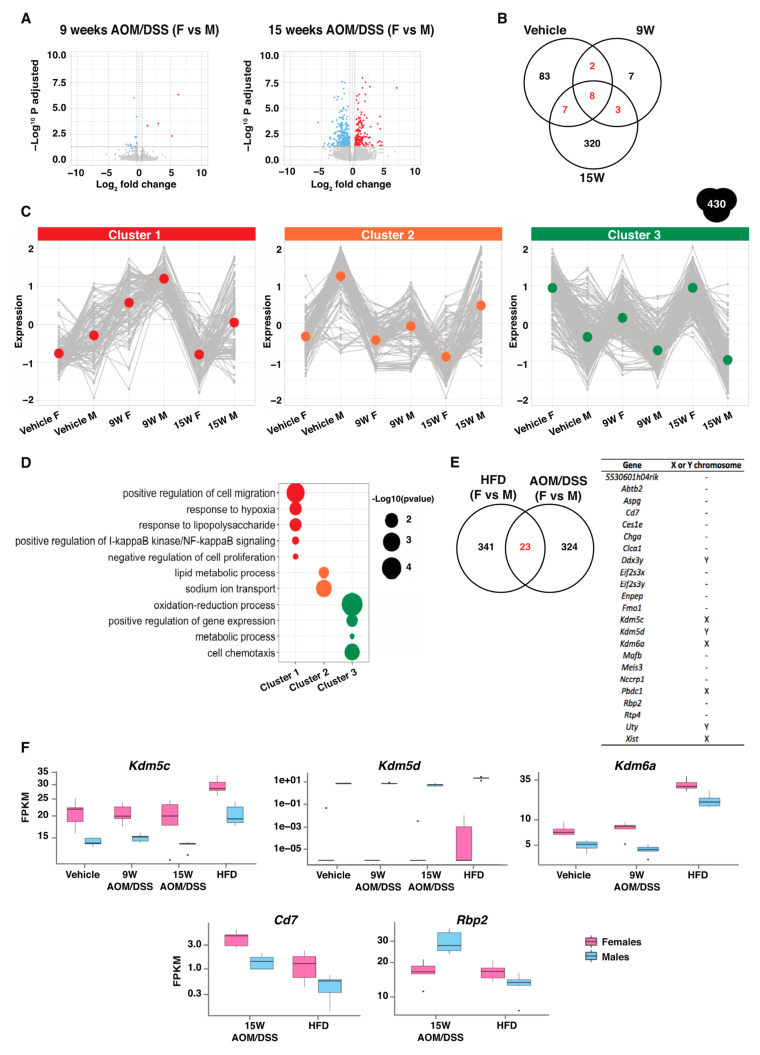
The largest sex differences were seen after 15 weeks of AOM/DSS treatment. (**A**) Volcano plot showing the DEGs between females and males during 9 and 15 weeks AOM/DSS treatment. (**B**) Venn diagram showing the common DEGs between the sexes during vehicle, and 9 and 15 weeks of AOM/DSS treatment, respectively. (**C**) The total 430 genes differentially expressed between the sexes were divided into three different clusters and the expression presented (mean centered with scaled FPKM values) in line plots. (**D**) The BPs separated based on cluster, with the size of the bubbles corresponding to the enrichment score (−log 10 (*p* value)). (**E**) Venn diagram comparing the DEGs between the sexes during AOM/DSS treatment and HFD feeding. (**E**) The 23 DEGs that are sex-specific regulations in both studies (**left**) and corresponding chromosomal location (**right**). (**F**) Boxplot showing the mRNA expression (FPKM) of the genes *Kdm5c, Kdm5d, Kdm6a, Cd7*, and *Rbp2* that were differentially expressed between the sexes during both AOM/DSS treatment and HFD feeding.

Next, instead of investigating the *response* to AOM/DSS, we directly compared the transcriptomes of the male colon with the female one. The sex differences during 9 weeks of AOM/DSS treatment were limited to only 20 DEGs (Figure 4A, Table S4), indicating that the transcriptomes of the two sexes became more similar at the state of colitis than at steady state (vehicle) or chronic inflammation (15 weeks). Still, sex differences in the oxidation-reduction process were also evident at 9 weeks of AOM/DSS treatment (Figure 1C and Figure S3A). We identified a larger sex difference at 15 weeks of treatment, with 338 genes being differentially expressed (Figure 4A, Table S5). In addition to the oxidation-reduction process, these were also involved in the metabolic process, positive regulation of T cell proliferation, and cellular response to interferon-gamma (Figure S3A). In total, 430 genes were differentially expressed between the sexes in at least one condition (Figure 4B,C). We divided these 430 genes into three clusters (Figure S3B), and their expression is presented in line plots in Figure 4C. We identified three sets of gene clusters with higher expression in males compared to females (Figure 4C). Genes in these clusters were involved in response to hypoxia and lipopolysaccharides, NFκB signaling, and negative regulation of cell proliferation (cluster 1), lipid metabolic process and sodium ion transport (cluster 2), and oxidation-reduction process, cell chemotaxis, and metabolic process (cluster 3), respectively (Figure 4D).

We also assessed this comparison (direct males-females comparison of AOM/DSS-colon transcriptomes) for similarities with the sex differences following HFD feeding in mice (from [12]). Interestingly, 23 DEGs were differentially expressed between the sexes in both experiments (Figure 4E, left). Seven of these are located on the X or Y chromosome (Figure 4E, right), including *Kdm5c*, *Kdm5d*, and *Kdm6a*. Two of these (*Kdm5c* and *Kdm6a*) were higher expressed in females and one (*Kdm5d*) in males, and these displayed consistent differences during all conditions (vehicle/normal, 9- and 15-week AOM/DSS treatment, and HFD, Figure 4F). Other genes, not located on sex chromosomes, also displayed sex differences during both 15-week AOM/DSS treatment and HFD. These included the T cell antigen *Cd7* and the retinol-binding protein 2 (*Rbp2*) (Figure 4E,F). The *Cd7* displayed a higher expression in females during both conditions, whereas *Rbp2* was significantly higher in males during AOM/DSS treatment but in females during HFD feeding (Figure 4F).

Overall, our data demonstrate significant sex differences in the colon transcriptome in basal gene expression (normal/vehicle) as well as during AOM/DSS treatment, especially after 15 weeks of treatment.

### 2.6. Sex Differences over the Course of Acute-to-Chronic Inflammation

**Figure 5:**
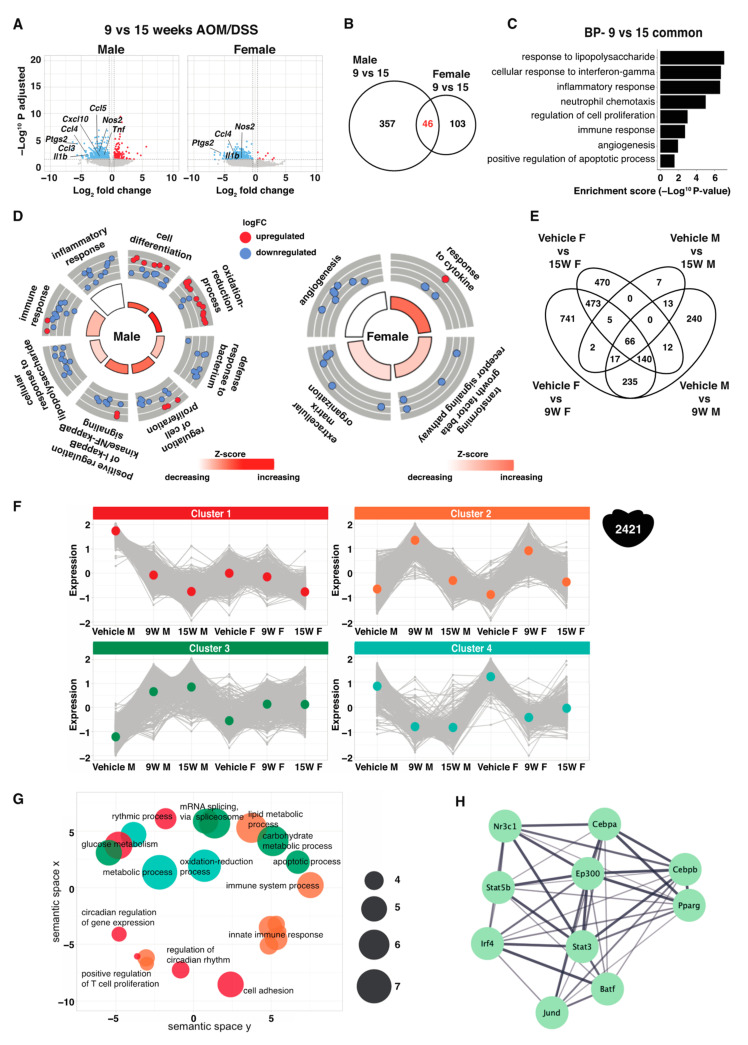
Sex differences in the response to acute and chronic inflammation. (**A**) Volcano plot showing DEGs between 9 weeks and 15 weeks of AOM/DSS treatment. (**B**) Venn diagram illustrating common and sex-specific responses after 15 weeks of AOM/DSS treatment compared to at 9 weeks. (**C**) BP enrichment analysis of the common and (**D**) sex-specific DEGs between 9 and 15 weeks of AOM/DSS treatment. (**E**) Venn diagram illustrating the 2421 total DEGs between vehicle treated and following 9 weeks of and 15 weeks AOM/DSS treatment in both sexes. M for male, F for female. (**F**) The 2421 DEGs were divided into four different clusters and their expression is presented as mean-centered and scaled FPKM values in line plots. (**G**) Corresponding BPs separated based on cluster in semantic space. The size of the bubbles corresponds to the enrichment score (−log 10 (*p* value)). (**H**) Transcription factors predicted with BART for the DEGs between 9 and 15 weeks of AOM/DSS treatment.

Upon further investigation of the transcriptomic differences between the more acute inflammation (9-week time point) and the more chronic inflammation (15-week time point), we noted that genes were downregulated (in both sexes) at 15 weeks compared to at 9 weeks. This included many genes involved in inflammation (Figure 5A) and can be indicative of the less acute phase at 15 weeks. Interestingly, there were major sex-specific changes, and only 46 genes were altered (all downregulated) in both males and females (Figure 5B and Figure S4A). These were involved in response to lipopolysaccharide and interferon-gamma, inflammatory and immune response, neutrophil chemotaxis, cell proliferation, apoptosis, and angiogenesis (Figure 5C). The male-specific genes were involved in immune and inflammatory response, defense response to bacterium, lipopolysaccharide signaling, NFκB signaling, and cell proliferation, which were all downregulated at 15 weeks (Figure 5D). The female-specific genes were involved in angiogenesis and were also downregulated at 15 weeks (Figure 5D).

A total of 2421 genes were regulated at least at one time point and in either sex in mice that underwent AOM/DSS treatment (Figure 5E). These genes were divided into four clusters depending on their pattern of regulation (Figure S4B) and presented as line plots in Figure 5F. The corresponding clusters present the dynamic changes during acute and chronic inflammation in males and females. Cluster 1 illustrates a set of genes that are downregulated during the course of the treatment in both sexes, but where the pattern starts from a higher level in males and exhibits an enhanced male-specific downregulation at 9 weeks. Cluster 2 illustrates a set of genes that were upregulated in mice of both sexes following 9 weeks of AOM/DSS and then returned to near-baseline levels after 15 weeks. Cluster 3 contains a set of genes that were also upregulated after 9 weeks in both sexes (although more in males) and then remained high at 15 weeks. The last cluster, cluster 4, contains a set of genes that were downregulated after 9 weeks of treatment in both sexes, and whose expression remained low at 15 weeks. Functional annotation of corresponding genes shows that cluster 1 was involved in cell adhesion and circadian rhythm, cluster 2 in inflammation-related pathways, cluster 3 in apoptosis and mRNA splicing, and cluster 4 in metabolic processes (Figure 5G and Figure S4C). Notably, cluster 2 (upregulated after 9 weeks and downregulated to basal levels after 15) included numerous inflammation-related genes (*Cxcl1, Cxcl2, Cxcl3, Cxcl5, Cxcl9, Cxcl10, Ccl3, Ccl4, Ccl5, Ccl8, Ccl20, Anxa1, Il1a, Il1b, Nfkbiz, Nos2, Ptgs2* (*Cox2*), *Rel*, and *Tnf*), which is in agreement with the more acute phase of inflammation at the 9-week timepoints. We finally used BART to predict transcription factors that regulate the genes in this cluster (#2) and identified Nr3c1 (Gr), Pparg, Stat3, Irf4, and Jund as predicted transcription factors (Figure 5H). Again, our data demonstrate that females and males present a sex-specific response to inflammation and highlight Stat3 and Gr as sex-specific regulators.

## 3. Discussion

Sex differences exist not only in reproductive organs but in many cell types, tissues, and organs and across species (reviewed in [15]). The underlying cause for sex differences can be related to size differences (males are often larger), sex chromosomes, sex hormones, genetic architecture, environment, or behavioral patterns. In the Drosophila melanogaster model, sex difference in intestinal stem cell proliferation has been reported, with the physiological consequence of a larger intestine in females [16]. However, the mechanism for most sex differences in non-reproductive organs remains unclear. Animal experiments enable a highly controlled environment, where sex differences to a large extent can be separated from behavioral differences. In mouse models, we and others have identified a sex-difference in terms of number and size of adenomas of the colon following AOM/DSS treatment, and of inflammatory markers [14,17]. We have also recently identified transcriptomic sex differences in the colon of mice, at the steady-state level (control diet) and in response to HFD [12]. In order to improve our understanding of sex differences during colitis and the subsequent impact on cancer development, we here describe the detailed analysis of the colon epithelial (scrape) transcriptome of AOM/DSS-treated mice of both sexes. AOM/DSS is known to induce inflammatory processes and expression of inflammatory genes such as *Cxcl1*, *Ccl4*, *Il1a, Il1b, Nfkbie, Nos2, Ptgs2 (Cox2)*, and *Tnf* [18]. We could confirm the regulation of these genes and establish that our experiment (AOM/DSS treatment, approach, and transcriptome analysis) worked as expected.

We found that male mice displayed a stronger response to AOM/DSS treatment at both investigated time points. The male-specific response was in part related to NFκB signaling, which directs the inflammatory response. This is in line with our previous findings of a stronger upregulation of certain cytokines in males [14], which could be related to a lack of the tumor suppressive and anti-inflammatory effects of estrogen (reviewed in [19]). Interestingly, the male-specific DEGs identified were also enriched for functions related to the circadian rhythm. The intestinal circadian rhythm is important for a variety of biological processes including cell proliferation, intestinal permeability, and immune homeostasis, and its disruption can contribute to both IBD and CRC [20]. The circadian clock has previously been found to be deregulated in mice with DSS-induced colitis and Arntl (Bmal1) knockout mice present aggravated DSS-induced colitis [21]. Moreover, recent studies in IBD patients indicate that clock gene disruption is an early event in IBD, as the expression of clock genes was significantly lower also in the non-inflamed mucosa of IBD patients compared to healthy controls [22].

A perhaps unexpected finding was the notable difference in the regulation of multiple steroid hormone receptors. Such nuclear receptors are powerful regulators, often implicated in anti-inflammatory signaling, and the noted sex difference in their regulations upon inflammation (9 weeks) may be a major contributor to the higher sensitivity to colitis and predisposition for tumor development in males. Several nuclear receptors were specifically downregulated in males (Vdr, Lrh1/Nr5a2, Mr/Nr3c2, Rev-erbβ/Nr1d2, Car/Nr1i3, Esrrg, Lxrα/Nr1h3) and have indeed been demonstrated to regulate key protective functions relating to colitis or CRC. This includes, for example, adherens junctions important for intestinal permeability (Vdr [23]), intestinal cortisol production (Lrh1 [24]), tumor-suppressive functions (Nr3c2 [25]), and suppression of CRC aggressiveness (Esrrg [26]). Importantly, Lxrα has been reported to inhibit proliferation of CRC cells and its overexpression to result in fewer and smaller intestinal tumors [27]. In relation to this, the female-specific upregulation of target genes of its homologue Lxrβ is interesting, as Lxrβ has also been demonstrated to protect against colitis [28]. Only two nuclear receptors were specifically upregulated in males (Pparg and Nur77/Nr4a1). In humans, PPARg polymorphism was recently reported to be associated with an increased risk of developing CRC [29]. Further, Nur77 is known to be expressed in macrophages and to play a role in inflammation, but it has also been reported to increase colon cancer progression in a crosstalk with TGFβ [30].

Our findings indicate that the male-specific AOM/DSS-induced genes were predicted to be regulated by Stat3. We also found support for Stat3 in sex-specific regulations of both an HFD-induced colitis model and in a paired human CRC and normal colon comparison. Interestingly, Stat1 has been identified as a sex-specific tumor suppressor in CAC in mice [31]. Stat members can detect a variety of signals at the inner cell membrane and transduce them to affect gene regulation. Stat1 and Stat3 converge in regulating cytokine and growth factor receptors, but they play opposite roles in tumorigenesis and Stat3 is considered an oncogene [32]. Our finding that Stat3 has a sex-specific role in regulating male-specific genes in the colon may indicate that it contributes to the development of larger tumors in males during CAC. The female-specific AOM/DSS-induced genes, on the other hand, were predicted to be regulated by the nuclear receptor Gr (Nr3c1). The glucocorticoid receptor signaling is important to heal DSS-induced colitis [33] and potential female-specific activation of Gr may explain why males are more affected by AOM/DSS treatment. Further studies will be needed to understand and evaluate the further consequences of these specific sex differences.

In our study, we found that the non-coding RNA X-inactive specific transcript (*Xist*) was 12-fold higher expressed in the female colon (vehicle treatment). *Xist* has a central role in X chromosome inactivation and is only expressed in cells containing at least two X chromosomes (i.e., females) [34]. We also discovered specific and strong sex differences in the expression of three histone lysine demethylases, namely *Kdm5c, Kdm5d*, and *Kdm6a*. These genes are all located on the sex chromosomes, and the Y-linked Kdm5d was recently reported to be sex-dependently upregulated also in male neural crest-derived stem cells in rats [35]. Deregulation of lysine demethylases is frequently observed in cancer [36], and may thus also contribute to sex differences in tumorigenesis. However, genes not expressed on the sex chromosomes were also significantly differentially expressed between the sexes, as demonstrated in our study. Another histone demethylase, retinol-binding protein 2 (*Rbp2*, at chromosome 9), was specifically upregulated in males following AOM/DSS treatment (15 weeks), but in females following HFD. *Rbp2* has been reported to regulate gut endocrine signaling and body weight [37] which may be a reason for the different regulations in response to HFD (and resulting weight gain). This gene is also overexpressed in gastric cancers, where its inhibition triggers senescence of malignant cells and may be used as an anticancer strategy [38]. Interestingly, the T-cell antigen *Cd7* was significantly higher expressed in females (compared to males after 15 weeks of treatment) along with a general enrichment of genes involved in the regulation of T cell proliferation. Other studies have found sex differences related to regulatory T cells, including after ischemic stroke in mouse models [39], and in human blood [40,41,42].

We also identified a sex-dependent transcriptomic response along the course of AOM/DSS treatment (9 and 15 weeks). As expected, many inflammatory genes were first strongly upregulated (9 weeks) to later become less expressed or resemble basal levels (15 weeks), and the response overall was stronger in males. We note that after 9 weeks of treatment, males showed an activation of the adaptive immune response, whereas females showed an activation of the innate immune response, based on functional annotation of regulated genes. After 15 weeks of treatment, females also showed an activation of the adaptive immune response, although the specific genes were not the same as in males. It is possible that males and females have different kinetics in their response to AOM/DSS treatment and/or activation of the adaptive immune response. Based on their disease activity index (DAI) curve previously generated for these mice, [14] females presented a quicker response to DSS-induced colitis and a faster resolution after DSS treatment. In line with this, Rathod et al. found that females are protected against systemic inflammation-induced endothelial dysfunction, likely due to the accelerated resolution of inflammation in females compared to males [43]. It is possible that the female-pronounced Gr activity, as indicated by our study, contributes to their faster resolution of inflammation.

To be noted, while our study can help understand aspects of sex differences in colitis, IBD, and CRC development, the results may not mimic the human situation. It is important to consider potential species differences as well, in addition to the cause of inflammation in this mouse model being different. Here, mucosal trauma is induced by a chemical agent (DSS) which leads to colitis, whereas in IBD, an antibody-antigen reaction against the mucosa of a susceptible individual causes the inflammation.

Altogether, our data clearly demonstrate, for the first time, significant and intriguing sex differences in the transcriptomic response to colitis. Most interestingly, we describe how males increase inflammatory signaling and adaptive immune cell signaling, have a more deregulated circadian rhythm, and activated Stat3-regulation, whereas females showed increased activation of the innate immune response and of Gr-mediated signaling. In summary, this study enables new insights into sex-specific gene expression in the colon. It also stresses the importance of including both sexes in studies of diseases while exploring fundamental mechanisms.

## 4. Materials and Methods

### 4.1. Animal Experiment

The animal experiment and outcomes have been previously described [14]. In short, 5 to 10-week-old C57BL/6J male (average body weight 23.6 g +/−0.3) and female (average body weight 18.3 g +/−0.2) mice were randomly assigned to treatments with either AOM (10 mg/kg body weight, i.p.) followed by three cycles of DSS (2.5%) in drinking water, or vehicle (0.9% NaCl, i.p.) for 9 or 15 weeks (Figure 1A). The clinical severity of colitis was evaluated by a disease activity index (DAI). Colons were harvested, and the number and size of intestinal adenomas were recorded. Tissues were prepared for histologic analyses and immunohistochemistry or snap frozen (incl. isolated colon epithelial-layer scrape) in liquid nitrogen for quantitative reverse-transcription PCR (qPCR). Hematoxylin and eosin (H&E) sections were examined and scored for colitis by a pathologist blinded to the mouse genotype. Raw data from the previous study were compared according to sex here and included in Figure 2A,B and Figure 3A. The epithelial layer of colon tissue from males and females was used for sequencing. Samples following treatment with vehicle (i.p. at week 0, sacrifice at week 9), and AOM/DSS (i.p. at week 0, DSS treatment cycles during weeks 1, 4, and 7, and sacrificed at week 9 and 15, see Figure 1A), with 5 biological replicates for each condition and sex (*n* = 5 per group) were used for sequencing (*N* = 30). The local ethical committee of the Swedish National Board of Animal Research approved all experimental protocols for the study (ID211/16).

### 4.2. RNA Isolation

The colon epithelial layer was physically separated from the lamina propria by scraping with a sharp object and snap frozen. The cells were homogenized using a tissue lyser (Qiagen, Chatsworth, CA, USA) and total RNA was isolated using QIAzol and miRNeasy Mini Kit (Qiagen, Chatsworth, CA, USA) with on-column DNAse treatment according to the manufacturer’s instructions. Quantitative and qualitative analyses of the RNA were performed with NanoDrop 1000 spectrophotometer and Agilent Tapestation 2200 (Agilent Technologies, Palo Alto, CA, USA), respectively. All samples had RNA integrity (RIN) >7.

### 4.3. RNA-Seq and Bioinformatic Analyses

Library preparation was generated with 200 ng total RNA as input (Illumina TruSeq Stranded mRNA) and sequencing (Illumina NovaSeq6000) was performed at Sweden’s National Genomics Infrastructure (NGI). Phred scores for all bases for each sample were between 30 and 40, indicating that the base call accuracy is above 99.9% (Figure S1A). The paired-end reads (2 × 51 bp in length) generated for each sample (at least 12 M) were mapped against the mouse genome (GRCm38) using STAR. The average number of sequenced reads was 34 M per sample and 72% of reads, on average, were duplicated (Figure S1B). Although the level of duplicate reads was high, about 10 M unique reads for each sample remained, which was sufficient for an analysis of good quality. FeatureCounts and StringTie were used to generate gene counts and TPM values. DESeq2 was used to calculate differentially expressed genes (DEG) with raw counts as input and the Benjamini–Hochberg procedure was used to estimate the false-discovery rate (FDR). Genes were considered as significantly differentially expressed if their FDR-adjusted *p*-values were < 0.05 and the log 2 FC > |0.4|. Cut off for non-expressed genes was <1 FPKM in both compared groups. The RNA-seq data is uploaded at GEO with accession nrGSE201360. Gene ontology/biological function was performed using the DAVID bioinformatics website. The GOCircle and GOChord plot were done in R (version 3.6.3) using GOplot (version 1.0.2). The GOCircle plot was used to demonstrate the gene expression and gene-annotation enrichment data of the BPs. The GOChord plot was used to assign specific genes to their assigned BPs. Transcription factors were predicted using the web server BART (version 2.0). BART uses over 5000 public mouse ChIP-seq datasets to predict those that bind to cis-regulatory chromatin regions and regulate the expression of the identified gene. The transcription factors and their interactions were visualized using STRING (version 11.0). Factoextra (version 1.0.7) and k-means cluster analysis with fviz_cluster in R (version 3.6.3) was used to divide the genes into different clusters based on their dissimilarity. The REVIGO web server [44] was used to summarize long lists of GO terms by finding representative subsets of terms based on a clustering algorithm that uses semantic similarity measures. Transcriptomic signal-to-noise ratio (tSNR, i.e., the ratio of divergence between the mean and replicate variability) was calculated with Euclidean distance metric in R. Variance-stabilized counts were used as input.

## Data Availability

Gene expression data are deposited in the NCBI Gene Expression Omnibus database (GSE201360).

## Supplementary Materials

The following supporting information can be downloaded at: https://www.mdpi.com/article/10.3390/ijms231810408/s1.
