# Supplementary material for: Colitis Induces Sex-Specific Intestinal Transcriptomic Responses in Mice
Source: Int J Mol Sci. 2022 Sep 8;23(18):10408. doi: 10.3390/ijms231810408 (PMC9499483; doi:10.3390/ijms231810408)
Supplement: Supplementary file 1 [file ijms-23-10408-s001.zip › ijms-1887041-supplementary figures.pdf]

Supplementary information for

Sex-Specific Transcriptomic Response to AOM/DSS-Induced Colitis and Colitis Associated Colorectal Cancer

Linnea Hases, Madeleine Birgersson, Rajitha Indukuri, Amena Archer and Cecilia Williams

Corresponding author: Cecilia Williams
Email: cecilia.williams@scilifelab.se

This PDF file includes:
Figure S1: RNA-sequencing quality.
Figure S2: Transcription factors predicted with BART analysis in both mouse and human.
Figure S3: Sex differences in the transcriptome during vehicle, 9 weeks, and 15 weeks AOM/DSS treatment.
Figure S4: Transcriptomic differences between 15 and 9 weeks AOM/DSS treatment.

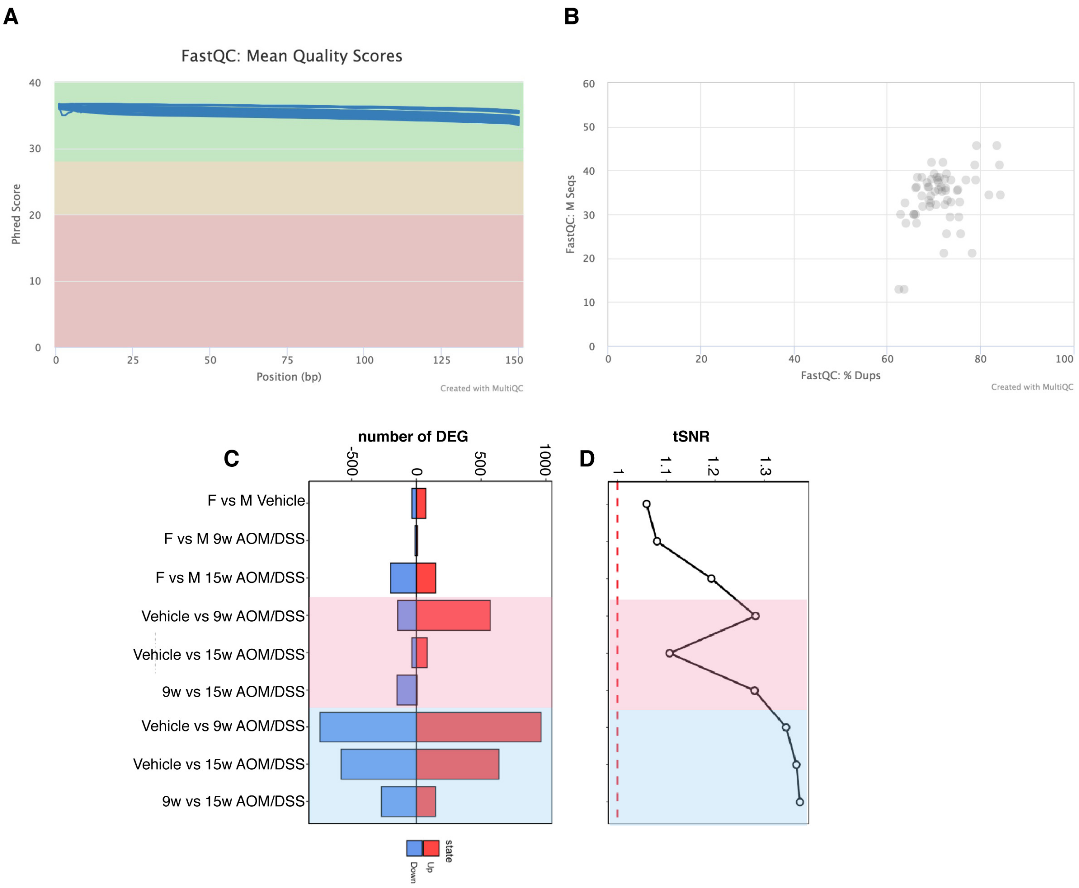

Figure S1. Good quality of the RNA-sequencing. (A) The mean quality score was between 30 and 40 for all 150 bp for all samples sequenced. **(B)** The number of million sequenced reads plotted against the percentage of duplicates. **(C)** The number of upregulated and downregulated differentially expressed genes (DEG) between all different comparisons. **(D)** The transcriptomic signal to noise ratio (tSNR) was larger than one for all the comparisons tested.

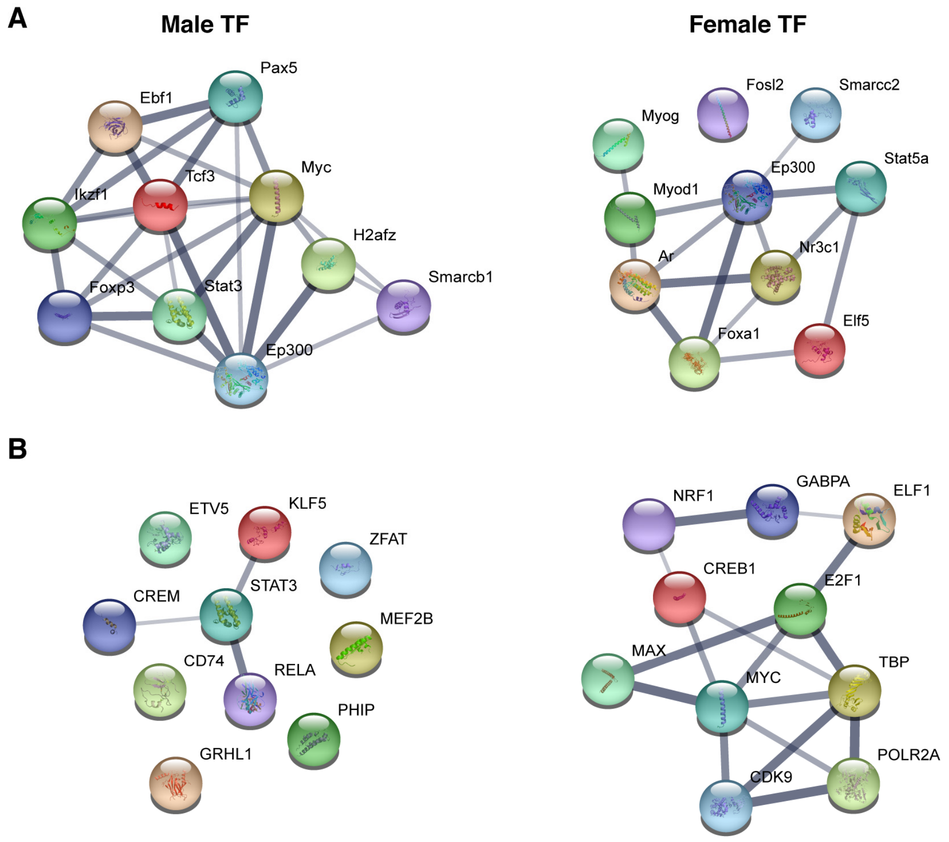

Figure S2. Transcription factors predicted with BART analysis in both mouse and human. (A) Transcription factors (TF) predicted with BART analysis in a high-fat diet (HFD) induced colitis model (compared to control diet) in males and females and **(B)** in human colorectal cancer (CRC) compared to normal colon.

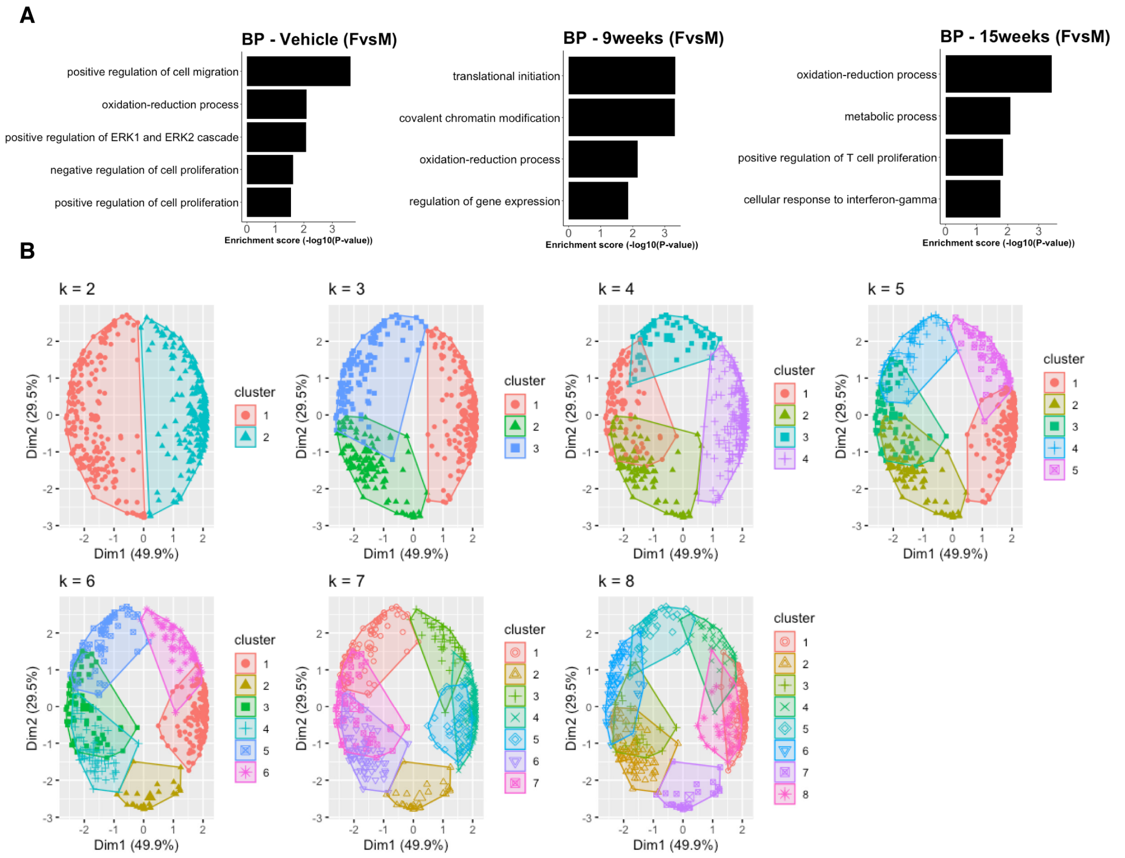

Figure S3. Sex differences in the transcriptome during vehicle, 9 weeks, and 15 weeks AOM/DSS treatment. (A) Biological process enrichment analysis for the DEG between sexes in vehicle, 9 weeks, and 15 weeks AOM/DSS treatment. **(B)** K-mean clustering plots (k=2 to k=8) of genes DEG between sexes. k=3 showed the clearest separation between genes.

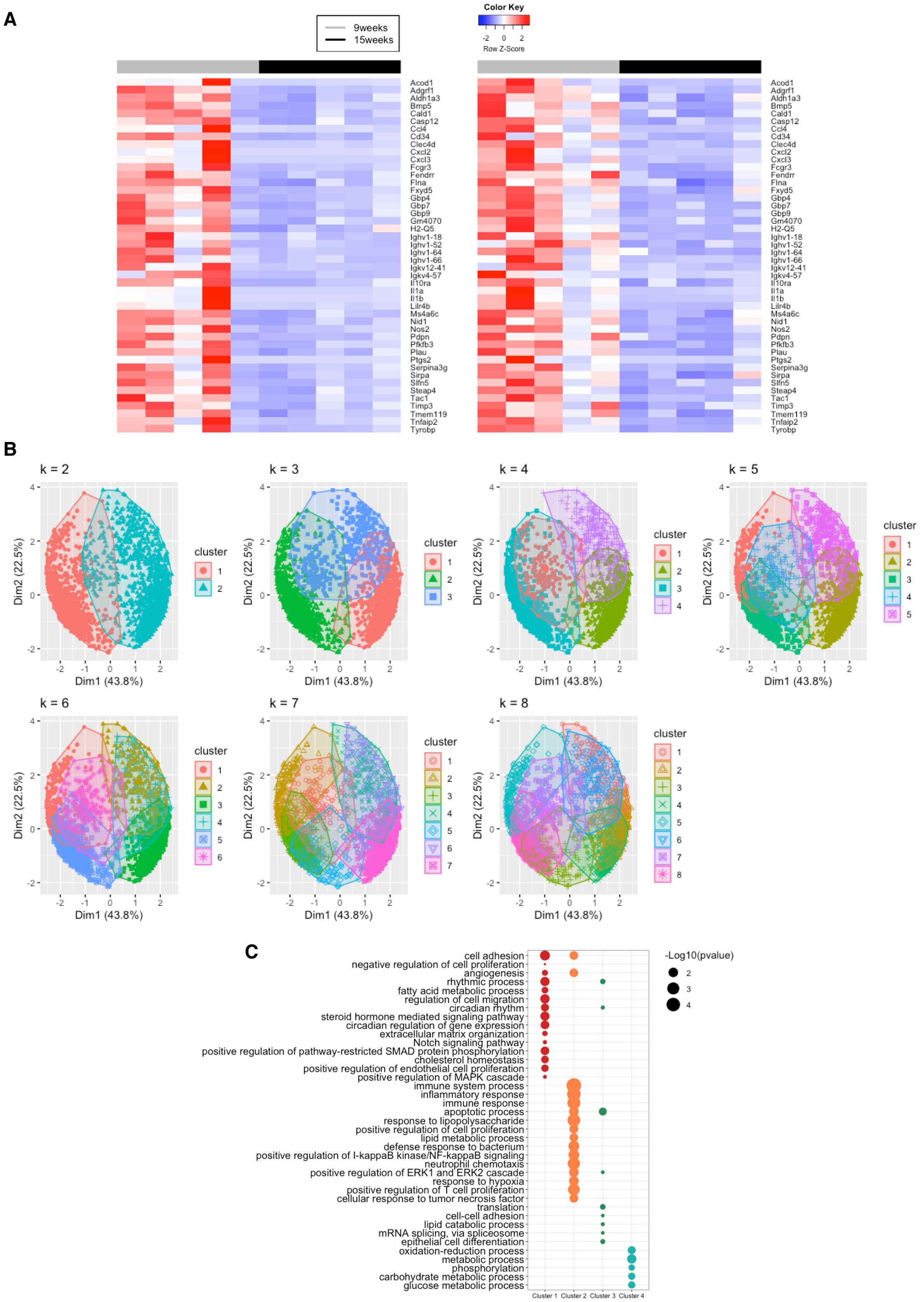

Figure S4. Transcriptomic differences between 15 and 9 weeks AOM/DSS treatment. (A) Heatmap of the common DEG in females (left) and males (right) comparing 9 week- and 15 week-AOM/DSS treatment. **(B)** K-mean clustering plots (k=2 to k=8) of genes DEG between vehicle and AOM/DSS treatments in both sexes. k=4 showed the clearest separation between genes. **(C)** BPs separated based on the four clusters, the size of the bubbles corresponds to the enrichment score (-log10(Pvalue)).
